# Evaluation of a universal long-lasting insecticidal net (LLIN) distribution campaign in Ghana: cost effectiveness of distribution and hang-up activities

**DOI:** 10.1186/1475-2875-13-71

**Published:** 2014-02-28

**Authors:** Lucy Smith Paintain, Elizabeth Awini, Sheila Addei, Vida Kukula, Christian Nikoi, Doris Sarpong, Alfred Kwesi Manyei, Daniel Yayemain, Etienne Rusamira, Josephine Agborson, Aba Baffoe-Wilmot, Constance Bart-Plange, Anirban Chatterjee, Margaret Gyapong, Lindsay Mangham-Jefferies

**Affiliations:** 1 Department of Disease Control, Faculty of Infectious & Tropical Diseases, London School of Hygiene & Tropical Medicine, Keppel Street, London WC1E 7HT, UK; 2 Dodowa Health Research Centre, Dodowa, Dangme West District, Ghana; 3 UNICEF Offices, 4-8th Rangoon Close, Accra-North, Ghana; 4 National Malaria Control Programme, Ghana Health Service, PO Box KB493, Accra, Greater Accra, Ghana; 5 Department of Global Health and Development, Faculty of Public Health and Policy, London School of Hygiene & Tropical Medicine, 15-17 Tavistock Place, London WC1H 9SH, UK

## Abstract

**Background:**

Between May 2010 and October 2012, approximately 12.5 million long-lasting insecticidal nets (LLINs) were distributed through a national universal mass distribution campaign in Ghana. The campaign included pre-registration of persons and sleeping places, door-to-door distribution of LLINs with ‘hang-up’ activities by volunteers and post-distribution ‘keep-up’ behaviour change communication activities. Hang-up activities were included to encourage high and sustained use.

**Methods:**

The cost and cost-effectiveness of the LLIN Campaign were evaluated using a before-after design in three regions: Brong Ahafo, Central and Western. The incremental cost effectiveness of the ‘hang-up’ component was estimated using reported variation in the implementation of hang-up activities and LLIN use. Economic costs were estimated from a societal perspective assuming LLINs would be replaced after three years, and included the time of unpaid volunteers and household contributions given to volunteers.

**Results:**

Across the three regions, 3.6 million campaign LLINs were distributed, and 45.5% of households reported the LLINs received were hung-up by a volunteer. The financial cost of the campaign was USD 6.51 per LLIN delivered. The average annual economic cost was USD 2.90 per LLIN delivered and USD 6,619 per additional child death averted by the campaign. The cost-effectiveness of the campaign was sensitive to the price, lifespan and protective efficacy of LLINs.

Hang-up activities constituted 7% of the annual economic cost, though the additional financial cost was modest given the use of volunteers. LLIN use was greater in households in which one or more campaign LLINs were hung by a volunteer (OR = 1.57; 95% CI = 1.09, 2.27; p = 0.02). The additional economic cost of the hang-up activities was USD 0.23 per LLIN delivered, and achieved a net saving per LLIN used and per death averted.

**Conclusion:**

In this campaign, hang-up activities were estimated to be net saving if hang-up increased LLIN use by 10% or more. This suggests hang-up activities can make a LLIN campaign more cost-effective.

## Background

Long-lasting insecticidal nets (LLINs) are one of the most efficacious preventive interventions against malaria morbidity and mortality available [1] and form a cornerstone of the Roll Back Malaria (RBM) Partnership’s scaling-up for impact strategy to reduce malaria-related mortality by 75% from 2000 levels by 2015 [2]. In recent years, substantial gains have been made in moving towards the goal of universal coverage, in large part due to mass campaign distributions through which hundreds of millions of LLIN have been distributed in sub-Saharan Africa since 2002 [3].

The current global financial crisis means that funding for future LLIN distributions is likely to be much more restricted than previously [3], and it will be more important than ever to ensure that LLINs are being delivered as efficiently and effectively as possible, and that people are using their nets for as long as they are viable. A reasonable evidence base exists for the cost effectiveness of LLINs delivered through campaigns targeted at biologically vulnerable groups (either stand-alone or integrated with other child health interventions) or continuous distribution channels such as antenatal or immunization clinics [4]. However, there are few published studies reporting the costs or cost effectiveness of universal mass campaigns [5].

Evaluations of the early campaigns found that use of LLINs tended to lag behind ownership [6]. Novel and more intensive sensitization activities are now being integrated in to mass campaigns such as house-to-house visits to ensure hang-up of campaign LLINs is completed and to encourage higher LLIN usage. It is recognized that inclusion of hang-up activities requires additional resources (both financial and human) and there is particular interest in the cost effectiveness of the ‘hang-up’ component of LLIN campaigns.

Between December 2010 and October 2012, Ghana Health Service (GHS) with support from UNICEF, DFID, GFATM and other partners distributed approximately 12.5 million free LLINs through a universal mass distribution campaign with hang-up activities in all ten regions of Ghana. The cost-effectiveness of the Ghana LLIN Campaign was evaluated using a before-after design from a provider and societal perspective, and the incremental cost-effectiveness of the hang-up activities was estimated to add to the evidence base for decision-making on future LLIN distribution strategies.

## Methods

### Implementation of the LLIN campaign

Activities which formed the core basis of the universal LLIN campaign in Ghana included pre-registration of persons and sleeping places, door-to-door distribution of LLINs by volunteers including hang-up activities, and post-distribution ‘keep-up’ behaviour change communication activities.

Trained volunteers registered the number of people and sleeping spaces in households in the districts to benefit from the Hang-Up Campaign. This was used as the basis for the allocation of the LLINs and related items. In the first instance, the number of LLINs was calculated as number of household members divided by two; if this was greater than or less than the number of sleeping spaces then the number of LLINs was adjusted to equal the number of sleeping spaces.

Trained volunteers were informed of the final LLIN allocations to households in their community and collected the LLINs from the nearest pre-positioning site. The volunteers then delivered the allocated number of LLINs to each household. Where they were permitted to enter, they helped the occupants to hang the nets above each sleeping space in the household. Where they were not permitted to enter, they were instructed to provide rope and nails. In both cases the volunteers also delivered behaviour change communication (BCC) messages about use.

The volunteers who conducted the household registration and hang-up were recruited from the communities in which they worked. They relied on local knowledge of community members and especially household heads to identify registered households for LLIN distribution. Each hang-up team (made up of three volunteers) had a member who had participated in the household registration in the area (s) where the team carried out the hang-up.

### Study setting

This paper focuses on evaluation of the LLIN Campaign in Brong Ahafo, Central and Western regions, which were selected for pragmatic reasons, based on timing of data collection relative to campaign implementation and funding support. The selected regions cover all three of Ghana’s ecological zones: Central and Western regions are in southern Ghana and include parts of the coastal and forest ecological zones; Brong Ahafo is in central Ghana and includes parts of the forest and savannah ecological zones. There are nevertheless socio-economic and cultural differences between these regions and those in the north or east of the country. Likewise, since the selection of Brong Ahafo, Central and Western regions for this evaluation was not random it is not possible to collate the regional results to produce a statistically representative national average. This would also be hard to interpret as universal campaign implementation was phased across the country, starting in Eastern region in December 2010 and ending in Greater Accra Region in October 2012.

### Overview of evaluation design

An uncontrolled before-after design was chosen for this evaluation, with attribution of effects of the LLIN Campaign through collection of data on source of nets owned by households and a thorough process evaluation [7]. A mixed methods approach was taken, involving quantitative pre- and post-campaign household surveys, post-campaign in-depth interviews and focus group discussions with key stakeholders, and a costing analysis.

Because the same implementation strategy for the campaign was used in all ten regions of Ghana, it was not possible to use a randomized controlled trial or even a controlled before-and-after design to allow for the individual components to be evaluated separately. Therefore, the incremental cost effectiveness of the ‘hang-up’ component was estimated using reported variation in the implementation of hang-up activities and LLIN use. To facilitate this comparison, detailed questions on exposure to each element of the campaign process were included in the post-campaign household survey questionnaire.

LLIN distribution in Central and Western regions took place in November-December 2011; distribution in Brong Ahafo was in May-June 2012. The post-campaign household survey was conducted in September-October 2012, approximately 11 months after LLIN distribution in Central and Western regions and five months in Brong Ahafo region.

### Collection of costs data

Financial and economic costs were collected from the societal perspective, meaning that direct and indirect costs to both LLIN providers and recipients were incorporated. An ingredients approach was used to identify all resources required to deliver LLINs through a mass universal campaign with hang-up activities [8].

Financial costs were obtained retrospectively from the financial reports and accounts of the implementation partners. Research and evaluation costs were not included. Costs were measured in Ghanaian Cedis (GHC) or United States Dollars (USD), depending on the currency of the original expenditure. Costs in GHC were converted to USD according to the average exchange rate for the year of the expenditure (1 USD equivalent to 1.42 GHC in 2010, 1.52 GHC in 2011, and 1.81 GHC in 2012). All costs were adjusted for inflation and are presented as 2012 USD using the consumer price indices available from the International Monetary Fund [9].

Economic costs recognize that the cost of using resources means that these resources are unavailable for productive use elsewhere, and include costs such as donated goods or volunteered time spent on the intervention. Information on the time that volunteers spent on campaign activities, what they would have been doing if not working on the campaign and estimates of their usual income was collected during focus group discussions. The time spent by GHS personnel and other partners on training and supervision for which they did not receive direct salary support was also valued and included as an economic cost.

Capital goods with an expected lifespan of more than one year were annualized using a discount rate of 3% according to the guidelines of the World Health Organization [10]. The lifespan of cars and motorbikes used in the campaign was estimated to be 5 years, based on information from UNICEF. An average useful lifespan of three years was assumed for the LLINs [3]. Other one-off costs of the campaign were also treated as capital costs, including sensitization, household registration and LLIN distribution and hang-up; essentially these are investments, which are expected to last as long as the useful LLIN lifespan. These costs were annualized across the average LLIN lifespan of three years using a discount rate of 3%. Consistent with the running costs of the UNICEF Ghana office, overheads were included at a fixed value of 7% of all financial provider-level expenditure. Economic costs are presented as the average annual economic cost over the effective lifespan of the LLIN.

Questions on whether the household had made some contribution to the volunteer for hang-up and the value of the contribution were included in the household survey questionnaire, along with questions to investigate the amount of time any member of the household had to wait at home for the LLIN hang up visit.

### Measurement of campaign effectiveness

The number of LLINs distributed in each region was obtained from GHS district reports. The effect of the campaign on LLIN ownership and use was measured using household surveys. Pre-campaign baseline data on LLIN ownership and use is provided at regional level by the Multiple Indicator Cluster Survey (MICS) conducted in September-December 2011 [11]. Post-campaign data on LLIN ownership and use was collected in Brong Ahafo, Central and Western regions in September-October 2012 using the same survey design and standardized questionnaire as MICS to allow direct comparison. Additional questions on households’ exposure to each phase of the LLIN Campaign were also included in the post-campaign questionnaire, including whether campaign LLINs were hung by the volunteer or not. Briefly, the post-campaign household survey followed a two-stage cluster sample design. At least 543 households were required in each of the three evaluation regions to give an estimate of the proportion of children under five who slept under an LLIN the night before the survey to within 8% precision (further details to be reported elsewhere; Awini *et al.,* personal communication).

Data processing and analysis was conducted in Stata 12.0, using ‘svy’ commands to ensure confidence intervals were appropriately adjusted according to the design of the surveys. Determinants of post-campaign LLIN use were explored using logistic regression; explanatory variables with a p-value of <0.1 in univariable analyses were included in the final multivariable model.

### Cost effectiveness analysis

The increase in number of individuals that slept under an LLIN was estimated using data from the pre- and post-campaign household surveys and population data from the 2010 Ghana Census for each region. The measures of effect were:

∎ *Additional number of persons using an LLIN* – difference between number of individuals sleeping under an LLIN pre-campaign and the number sleeping under an LLIN post-campaign.

∎ *Additional number of children under five years using an LLIN* – difference between the number of children under five sleeping under an LLIN pre-campaign and the number sleeping under an LLIN post-campaign.

∎ *Additional number of all-cause under five deaths averted* – estimated number of deaths averted each year from use of LLIN was predicted based on the change in the number of children under five years sleeping under an LLIN following the campaign and the pooled estimate of 5.5 child deaths averted per 1000 children protected by insecticide-treated nets (ITNs) [1].

For each of these outcomes, an incremental cost effectiveness ratio (ICER) was calculated i.e. the additional effect (person using an LLIN or death averted) for additional cost of the LLIN Campaign compared to no campaign.

### Sensitivity analyses

The cost analyses involve a number of important assumptions. To investigate the effect of these assumptions on the results, each was varied in turn in one-way sensitivity analyses. Estimates of the useful lifespan of an LLIN vary by setting, therefore a lower value of two years and upper value of five years were explored [3]. These changes in LLIN lifespan were applied to all annualized campaign costs. The financial cost of the LLINs decreased considerably over the period of the LLIN Campaign. Cost estimates of USD 3.25 and USD 4.80 per LLIN used in the sensitivity analysis were median 2012 prices for a rectangular net (as distributed in the LLIN Campaign) or a conical net, respectively [12]. To explore the importance of assumptions regarding the predicted mortality impact of the LLIN Campaign (such as the influence of transmission intensity), the upper and lower uncertainty limits for the pooled estimate of 5.5 child deaths averted per 1,000 children protected by an LLIN were used (95% CI: 3.39, 7.67) [1]. The discount rate was also varied with lower and upper values of 0% and 10% [13].

### Estimating the incremental cost-effectiveness of hang-up activities

The study design did not allow direct comparison of a universal LLIN campaign with and without hang-up activities. However, the effect of the hang-up was derived by comparing LLIN use in households in which volunteers had hung-up one or more LLINs with use in households where the volunteer did not hang the net (s). Costs were estimated of a universal campaign without hang-up visits to households. In this alternative scenario, LLIN distribution was conducted from a fixed-point without any household follow-up visits for hang-up: it was assumed that the same volunteers involved in household registration attended a fixed distribution point for three days to deliver nets to recipients; the number of volunteers needed for registration and fixed-point distribution is approximately one-third that for door-to-door distribution. No time was included for house-to-house visits by volunteers. Supervision of distribution was reduced from ten days to three days. All other costs were kept constant.

The effectiveness on LLIN use of this campaign strategy was assumed to be lower, based on data from the post-campaign household survey data which found that LLIN use amongst individuals and children under five living in households where all or some of the nets were hung by a volunteer was significantly higher than LLIN use amongst those living in households where nets weren’t hung by the volunteer.

Thus, the ICER of a campaign with hang-up activities compared to one without was calculated. The sensitivity of this predicted ICER to the assumption about the additional effect of hang up was explored by varying the odds ratio of LLIN use by children under five living in households where nets were or were not hung by a campaign volunteer.

### Ethical considerations

Approval for the evaluation was granted by Ghana Health Service. Ethics approval was granted by the Observational/Interventions Research Ethics Committee of the London School of Hygiene & Tropical Medicine. Appropriate authorities were informed in each region and district involved in the evaluation. Individual informed written consent to participate in the study was obtained from heads of households and key informants.

## Results

A total of 1,327,601 LLINs were delivered to households in Brong Ahafo, 996,023 in Central, and 1,340,404 in Western regions; in each region, this represented over 99% of the LLINs procured. Overall, 79.2% of households surveyed reported that they had received at least one LLIN from the campaign and of these 38.2% had all, 7.3% had some and 54.5% had none of their nets hung by a campaign volunteer. The most common reasons for the volunteer not having hung the net were concerns about privacy or lack of space. More details on the process evaluation will be reported elsewhere (Awini *et al.*, personal communication).

Volunteers received a daily payment of GHC 10 from GHS and their partners for the registration exercise; however insufficient funds were available to pay the trebled number of volunteers for the house-to-house distribution and hang-up. Although the original design of the campaign was to be at zero cost to recipients, it emerged that volunteer motivation was low and some communities agreed to make contributions to their volunteers. The household survey data found that 65% of households made some contribution to volunteers with a median of GHC 0.50 (approximately USD 0.30) per net.

### Financial and economic costs of the LLIN campaign

**Figure 1:**
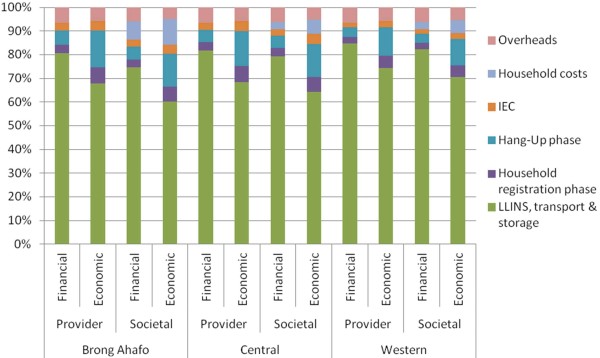
**Proportional distribution of cost per LLIN delivered across cost categories.** Distribution of costs across categories is presented by region and according to perspective of cost analysis.

The total financial cost of the campaign from the provider perspective across all three regions was USD 23.85 million (USD 6.78 million in Brong Ahafo region, USD 7.44 million in Central region, and USD 9.63 million in Western region); the average annual economic cost from the societal perspective was USD 10.64 million (USD 3.21 million, USD 3.34 million and USD 4.10 million in each region, respectively) (Additional file 1). LLINs, transport and storage comprise around 80% of the total financial cost (from both the provider and societal perspectives); this is reduced to around 65% of the annual economic cost once the costs associated with volunteer and household time have been included (combined figures provided in Table 1; regional breakdown presented in Figure 1).

**Table 1:** Distribution of financial cost and average annual economic cost per LLIN delivered by the Ghana LLIN campaign

	**Total financial cost**		**Average annual economic cost**	
	**USD (2012 prices)**	**%**	**USD (2012 prices)**	**%**
**LLINs, transport & storage**	**5.38**	**79.1**	**1.90**	**65.4**
LLINs	4.96	72.9	1.75	60.4
Non-LLIN materials	0.36	5.3	0.13	4.3
Transport & storage	0.06	0.9	0.02	0.7
**Household registration phase**	**0.21**	**3.1**	**0.17**	**5.7**
Regional planning & training of trainers	0.03	0.5	0.02	0.5
Training of volunteers	0.03	0.5	0.02	0.8
Registration exercise	0.09	1.3	0.10	3.3
Supervision	0.06	0.8	0.03	1.1
**LLIN distribution (including hang-up)**	**0.32**	**4.8**	**0.38**	**12.9**
Logistics training	0.05	0.7	0.02	0.7
Training of volunteers	0.10	1.5	0.06	2.1
Hang-up exercise	-	-	0.20	6.9
Supervision	0.17	2.6	0.09	3.3
**Information, education & communication**	**0.17**	**2.5**	**0.10**	**3.4**
Social mobilization	0.11	1.6	0.04	1.4
Keep-up planning & supervision	0.01	0.2	0.01	0.5
IEC materials & activities	0.05	0.7	0.04	1.6
**Overheads**	**0.43**	**6.3**	**0.15**	**5.3**
**Total provider cost**	**6.51**		**2.69**	
**Household-level costs**	**0.29**	**4.3**	**0.21**	**7.3**
Contribution to volunteers	0.29	4.3	0.10	3.5
Waiting time	-	-	0.11	3.7
**Total societal cost**	**6.80**		**2.90**	

Costs are presented in USD 2012 prices and summarized across all three regions (Brong Ahafo, Central & Western).

The financial cost per LLIN delivered was USD 6.51 from a provider perspective and USD 6.80 from a societal perspective, while the annual economic cost was USD 2.90 per LLIN delivered (societal perspective). Costs were much lower in Brong Ahafo than the Central or Western regions (regardless of perspective), which to a large extent reflects reductions in the purchase price of the LLINs over time: USD 3.40 per LLIN for Brong Ahafo in 2011, compared to USD 4.70 per LLIN in 2010 for the Central and Western regions (Table 2). Differences in the delivery cost per LLIN (i.e. excluding the cost of the LLINs) between Central and Western region (Table 2) are due to the additional LLINs delivered in Western region since the two regions had a similar number of volunteers involved in registration (approximately 3,500) and hang-up (approximately 11,000). Otherwise, the proportional distribution of total cost of the campaign between different cost categories was similar across the three regions (Figure 1, Additional file 1). A greater proportion of the economic cost is attributable to the registration and hang-up phases of the campaign (approximately 6% and 13%, respectively); this is due to the inclusion of the volunteers’ time and that of the personnel involved in training and supervision.

**Table 2:** Cost-effectiveness of the Ghana LLIN Campaign with door-to-door distribution and hang-up activities

	**Brong Ahafo**	**Central**	**Western**	**Combined**
Population^1^	2,278,862	2,115,757	2,303,207	6,697,826
Number of LLINs procured	1,338,000	1,003,100	1,346,900	3,688,000
Number of LLINs delivered	1,327,601	996,023	1,340,404	3,664,028
**Cost of LLIN Campaign (USD, 2012 prices)**				
Total financial cost (provider perspective)	6,784,971	7,437,331	9,625,732	23,848,034
Annual economic cost (societal perspective)	3,207,176	3,335,191	4,100,270	10,642,637
**Effect of LLIN Campaign**				
% of total population sleeping under an LLIN				
Pre-campaign	26.7%	16.8%	23.0%	21.3%
Post-campaign	68.5%	45.0%	46.9%	54.4%
% of children under five sleeping under LLIN				
Pre-campaign	42.0%	27.9%	32.7%	33.9%
Post-campaign	75.9%	53.4%	57.5%	63.0%
Additional number of persons using LLIN^1^	952,564	596,643	550,466	2,216,980
Additional number of children under five years using LLIN^1,2^	115,880	80,928	85,679	292,360
Estimated annual number of child deaths averted as result of increased LLIN use^3^	637	445	471	1,608
**Cost-effectiveness of LLIN Campaign**				
Financial cost^4^ per LLIN delivered	5.11	7.47	7.18	6.51
Economic cost^5^ per LLIN delivered	2.42	3.35	3.06	2.90
Financial delivery cost^4,6^ per LLIN	1.08	1.41	1.15	1.19
Economic delivery cost^5,6^ per LLIN	0.99	1.21	0.93	1.03
Economic cost^5^ per additional person sleeping under an LLIN	3.37	5.59	7.45	4.80
Economic cost^5^ per additional child under five years sleeping under an LLIN	27.68	41.21	47.86	36.40
Economic cost^5^ per child death averted	5,032.12	7,493.09	8,701.59	6,618.64

^1^Population estimates from 2010 census figures; ^2^Assumes 15% of population are under 5 years of age; ^3^Assumes 5.5 deaths averted per 1000 children under five years sleeping under an LLIN; ^4^Financial costs from the provider perspective; ^5^Average annual economic costs from the societal perspective (assumes 3-year useful LLIN lifespan; includes costs to recipients as well as providers); ^6^Excludes cost of LLIN and non-LLIN materials required for hanging. All costs presented in USD, 2012 prices.

### Effectiveness of the LLIN campaign

Significant improvements were achieved in LLIN ownership and use in all three regions that can be attributed to the LLIN Campaign. Household ownership of LLINs and use by all individuals, children under five years and pregnant women doubled between pre- and post-campaign household surveys. For example across the three regions, the proportion of households owning at least one LLIN increased from 42.5% pre-campaign to 85.3% post-campaign; the proportion of all individuals and children under five sleeping under an LLIN increased from 21.3% to 54.4% and 33.9% to 63.0%, respectively. Post-campaign LLIN ownership and use was higher in Brong Ahafo than Central and Western regions, although the relative improvements were similar across all three regions (Table 2).

Further analysis of the post-campaign survey data found that LLIN use by children under five was 77.4% amongst those living in households where some or all LLINs were hung by a campaign volunteer, compared to 53.9% in households which had not been assisted by a volunteer; thus hang-up by a volunteer increased the odds of a child sleeping under an LLIN by around 1.5 times when adjusted for other factors that may explain variation in use (adjusted OR: 1.57; 95% CI: 1.09, 2.27; p = 0.02) (Table 3).

**Table 3:** Logistic regression of the odds for LLIN use by children under five years after the Ghana LLIN Campaign

**Variable**	**N**	**%**	**Univariable analysis**			**Multivariable analysis**		
			**OR**	**95% CI**	**P-value**	**Adj OR**	**95% CI**	**P-value**
Area of residence								
Urban	274	50.2	1.00		<0.001	1.00		0.20
Rural	633	70.9	2.42	1.65, 3.54		1.35	0.85, 2.16	
Wealth quintile								
Poorest	188	81.6	1.00		<0.001	1.00		0.002
Second	272	69.3	0.52	0.33, 0.82		0.54	0.33, 0.89	
Middle	209	59.1	0.33	0.19, 0.57		0.38	0.20, 0.72	
Fourth	150	49.9	0.22	0.13, 0.39		0.32	0.17, 0.61	
Least poor	88	53.6	0.26	0.15, 0.46		0.61	0.30, 1.23	
Head of household has any education								
No	234	67.8	1.00		0.12			
Yes	671	61.7	0.76	0.54, 1.07				
Lives in HH with ≥1 LLIN observed hanging								
No	140	27.3	1.00		<0.001	1.00		<0.001
Yes	767	82.7	12.7	9.15, 17.7		10.0	6.98, 14.5	
Lives in HH with all/some LLINs hanged by volunteer								
No	474	53.9	1.00		<0.001	1.00		0.02
Yes	433	77.4	2.94	2.15, 4.01		1.57	1.09, 2.27	
Lives in HH with ≥1 LLIN per 2 people								
No	432	51.4	1.00		<0.001	1.00		<0.001
Yes	473	79.2	3.59	2.63, 4.91		3.33	2.31, 4.82	
HH respondent had heard ITN messages from community keep-up								
No	619	60.6	1.00		0.03	1.00		0.96
Yes	289	69.0	1.45	1.03, 2.03		0.99	0.69, 1.42	
HH respondent had heard ITN messages on radio								
No	518	61.4	1.00		0.23			
Yes	389	65.4	1.19	0.89, 1.58				
HH respondent had seen ITN messages on TV								
No	605	65.4	1.00		0.11			
Yes	302	58.7	0.75	0.53, 1.07				
HH respondent knew that ITNs prevent malaria								
No	201	53.7	1.00		0.001	1.00		0.06
Yes	706	66.3	1.70	1.24, 2.33		1.43	0.99, 2.06	

Odds ratios presented for Brong Ahafo, Central and Western regions combined.

The odds of sleeping under an LLIN were also significantly higher for children living in households with at least one LLIN observed hanging on the day of the visit, those living in households with at least one LLIN per two people and those living in the poorest households (Table 3).

### Cost per additional person and per additional child under five using an LLIN

The annual economic cost per additional person using an LLIN following the LLIN Campaign was USD 3.37 in Brong Ahafo, rising to USD 5.59 and USD 7.45 in Central and Western regions, respectively (Table 2). The higher costs in Central and Western regions compared to Brong Ahafo are due to a combination of the higher unit costs per LLIN delivered and the lower number of additional individuals using an LLIN as a result of the campaign.

A similar pattern was seen for the cost per additional child under five sleeping under an LLIN due to the LLIN Campaign: the economic cost in Western region (USD 47.86) was almost double that in the Brong Ahafo region (USD 27.68), with the cost in Central region somewhere in between (USD 41.21) (Table 2). This incremental approach assumes that if there had not been a mass universal campaign then LLIN use would have remained at pre-campaign levels and that there would be no costs associated with maintaining this level of LLIN use.

### Cost per additional death averted

At the LLIN use levels measured by the post-campaign survey, an estimated 1,608 child deaths were averted across the three regions in 2012 (637, 445, and 471 in Brong Ahafo, Central and Western regions, respectively). It is important to note that these deaths are additional to those that would have been averted with pre-campaign LLIN use i.e. are those directly attributable to the LLIN Campaign rather than the absolute number of deaths that could be averted by post-campaign LLIN use levels. The economic cost per child death averted was USD 6,619 (USD 5,032 in Brong Ahafo, USD 7,493 in Central and USD 8,702 in Western regions) (Table 2). Although the relative improvement in LLIN use by under-fives after the LLIN Campaign was similar across all three regions, a greater number of deaths are predicted to be averted in Brong Ahafo due to the higher overall proportions. In addition, because the overall costs for LLIN delivery were lower in Brong Ahafo, the financial and economic cost per death averted (whatever the perspective) was considerably lower in Brong Ahafo than Central and Western regions.

### Sensitivity analysis

**Figure 2:**
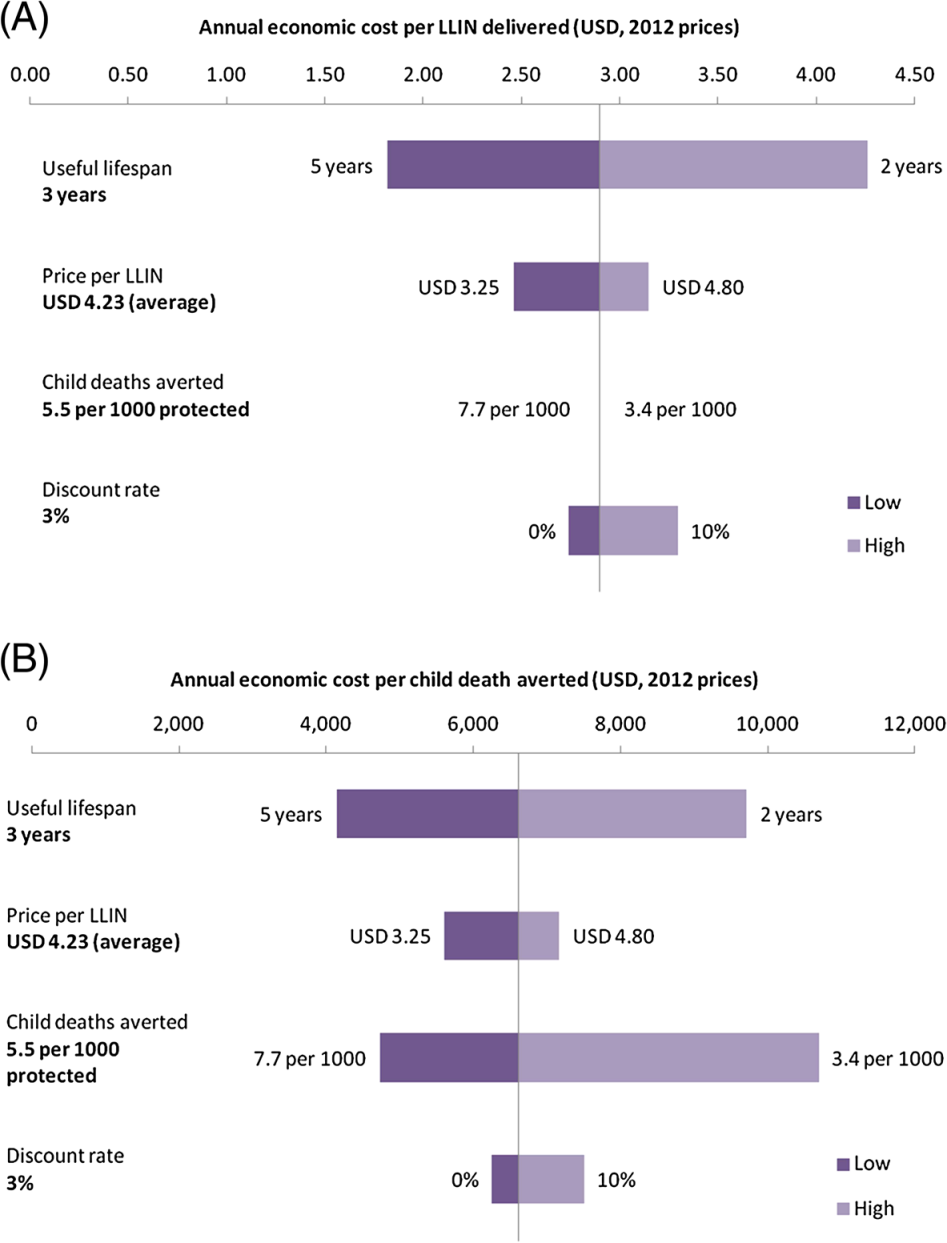
**Tornado diagrams showing sensitivity of ICERs to key assumptions. (A)** Cost per LLIN delivered; **(B)** Cost per child death averted.

The ICER was sensitive to useful lifespan of the net; a reduction in LLIN lifespan from three years to two years increases the cost per LLIN delivered and cost per death averted by around 50%. Conversely, if the lifespan of LLINs could be increased to five years then the cost per LLIN delivered and per death averted drop by around 40% (combined figures presented in Figure 2; regional breakdown presented in Additional file 2).

Using the median 2012 bulk purchase cost of a rectangular or conical net as the lower and upper unit costs showed the ICER was sensitive to the LLIN purchase price, and the regional differences reflect the variation in the price paid when procuring the nets. The ICER was very sensitive to the estimate of protective efficacy of the LLINs: if the lower uncertainty limit was used (3.4 deaths averted per 1,000 children protected by an LLIN), cost per death averted increased by around 60%; if the upper uncertainty limit was used (7.7 deaths averted per 1,000 children protected by an LLIN), cost per death averted decreased by around 30% (Figure 2; Additional file 2). The ICER was least sensitive to the choice of discount rate.

### Estimating the incremental cost-effectiveness of hang-up activities

In the comparison scenario of a universal campaign without hang-up visits to households, the number of volunteer-days and supervisor-days were reduced by replacing house-to-house distribution with fixed-point distribution and removing follow-up visits to households for hang-up. This reduced the financial provider-side costs by USD 0.10 (2%) per LLIN delivered. Although the direct financial savings were relatively modest, this alternative campaign design has the potential to substantially reduce the annual economic cost from the societal perspective by around 10%. This difference is because the unpaid time of volunteers is valued in the economic analysis so a reduction in the number of volunteer-days leads to a reduction in the overall full economic costs of the campaign.

However, the household survey data show that use was significantly higher in households where all or some of the campaign LLINs were hung by the volunteer (Table 3). Therefore, in the campaign delivery scenario in which no hang-up visits were conducted, the use by children under five that could have been achieved was adjusted accordingly. By reducing the proportion of children under five sleeping under an LLIN post-campaign in this scenario, the number of all-cause child deaths that could have been averted was lower and therefore the financial cost per child death averted increased by approximately 40%; the annual economic cost per child death averted also increased by approximately 30%.

**Figure 3:**
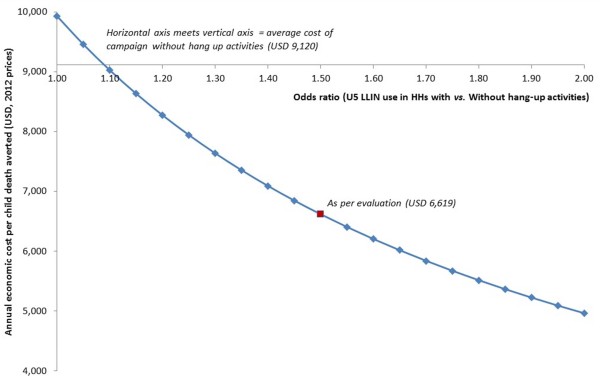
Sensitivity of ICER estimates of a universal LLIN campaign without hang-up activities to variations in the influence of LLIN hang-up on use by children under five.

The sensitivity of the predicted ICER (of a universal LLIN campaign with hang-up activities compared to one without) to the assumption about the additional effect of hang-up was explored. As the odds of a child under five using an LLIN in a household where LLINs had been hung by a volunteer increased, the cost per death averted decreased. A universal campaign with hang-up activities dominates a universal campaign without hang-up activities at an odds ratio of approximately 1.1 or greater (Figure 3). Thus, a minimum additional effect of hang-up activities of a 10% increase in under-five LLIN use is needed to achieve a cost-saving of a universal campaign with hang-up compared to a universal campaign without hang-up in this setting.

## Discussion

**Figure 4:**
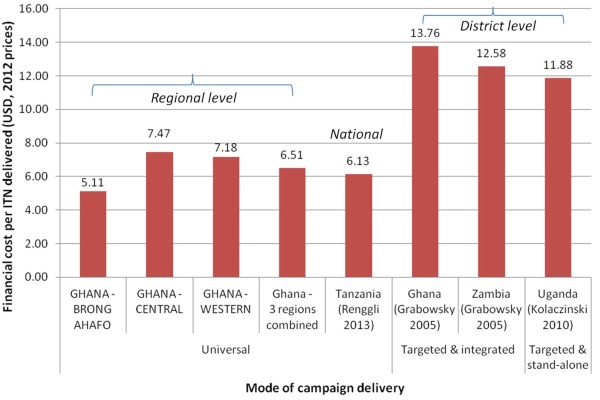
**Comparison of financial cost per LLIN delivered via different mass LLIN campaigns.** All financial costs presented from the provider perspective in 2012 USD.

The cost per LLIN delivered varied between the three regions and was considerably lower in Brong Ahafo than Central and Western regions. This was largely due to the reductions in LLIN procurement cost over time which resulted in lower purchase price of LLINs distributed in Brong Ahafo. Nevertheless, the financial cost per LLIN delivered through the universal mass campaign with hang-up activities in Ghana is comparable to other campaign distributions implemented using different delivery strategies, in different settings and at different scales (Figure 4). For example, the financial cost per LLIN delivered by the Ghana LLIN Campaign was comparable to that reported by the evaluation of another recent national universal campaign conducted in Tanzania in 2010–11 at around USD 6.10 per LLIN delivered [5]. When adjusted for inflation to 2012 USD, the financial cost per LLIN delivered by earlier targeted campaigns in Ghana, Zambia and Uganda was approximately twice as high as that found for the Ghana LLIN Campaign at around USD 12.00 [14-16]. It is important to note that these earlier campaigns in Ghana, Uganda and Zambia were conducted and evaluated at much smaller scale.

The annual economic cost per LLIN delivered by the Ghana LLIN Campaign was lower than the financial cost if it is assumed that all financial costs are incurred in year one of implementation. Thus, treating the campaign as an investment where the costs and effects are averaged across the expected useful lifespan of the LLINs makes the campaign even more cost effective.

Evaluating full economic costs from the societal perspective confirmed the importance of the unpaid time that volunteers committed to the campaign as well as contributions made by 60-75% of households, which helped to provide some level of compensation to the volunteers. The qualitative analysis (reported in detail elsewhere) also found that the level of support volunteers received from their community and supervisors was crucial to their motivation which in turn was an important facilitating factor in determining the success of the campaign (Awini *et al.*, personal communication).

**Figure 5:**
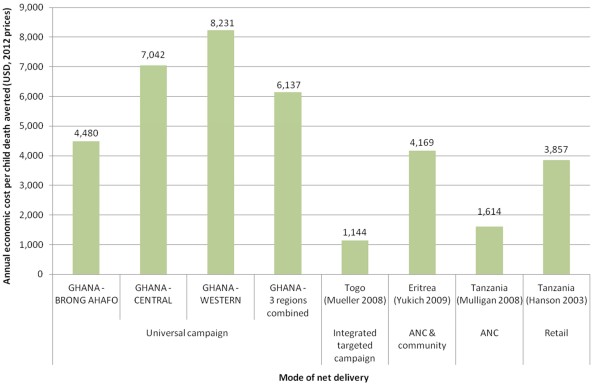
**Comparison of economic cost per death averted by different ITN distribution channels.** All economic costs presented from the provider perspective in 2012 USD.

Comparing the economic cost per child death averted with the results of other cost effectiveness evaluations of LLIN/ITN distributions is challenging as the results are highly variable (Figure 5). The cost per all-cause death averted in Brong Ahafo is comparable to the findings from community distribution in Eritrea [17] and social marketing in Tanzania [18]. However, the costs per death averted by the LLIN Campaign in Central and Western regions are considerably higher, particularly than those found for LLIN delivery through an integrated mass campaign in Togo [19] and the ANC-delivered voucher scheme in Tanzania [20]. The reason for these differences is not certain, although may relate to the outcome measure being additional deaths averted due to the campaign; the baseline ITN coverage in the earlier studies was much lower than that for the LLIN Campaign in Ghana and so relatively more additional deaths could have been prevented. Additionally, cost sharing is likely to have had some influence. For example, costs of the targeted campaign LLIN distribution in Togo were shared with the measles vaccination with which it was integrated [19].

As with previous studies, the cost estimates per LLIN delivered and per death averted by the Ghana LLIN Campaign were very sensitive to the purchase cost of the LLINs and the assumed useful LLIN lifespan [4]. Prices for bulk purchase of LLINs have reduced considerably over recent years and are currently around USD 3.25 for a rectangular net; at these prices, the annual economic cost to providers of the LLIN Campaign would have been around 20% less in Central and Western regions. Concerns that LLINs may have a shorter lifespan than three years have been raised [3] and this could increase annual economic cost per LLIN delivered by around 45%; conversely if technology development by LLIN manufacturers manages to increase LLIN lifespan to five years then the annual economic unit costs will decrease by a similar magnitude. In any case, monitoring of LLIN durability under field conditions is important to inform programmatic decisions regarding optimal LLIN distribution strategies [3]. The relative reduction in all-cause mortality that can be achieved by LLINs is likely to differ across different malaria transmission settings, hence the implications of using the lower and upper uncertainty limits around the 5.5 deaths averted per 1000 children protected by an ITN (95% CI: 3.4, 7.7) were explored [1]. The cost estimates per death averted were very sensitive to the assumed protection provided by LLINs, increasing by approximately 60% at the lower level of protective efficacy and decreasing by around 30% at the upper limit.

The incremental cost-effectiveness of the hang-up activities was explored by estimating the costs and effects of a universal campaign without hang-up activities. Although the difference in direct financial cost was relatively modest, this alternative campaign design reduced the annual economic cost per LLIN delivered by around 10%. This difference is because the unpaid time of volunteers is valued in the economic analysis so a reduction in the number of volunteer-days leads to a reduction in the economic costs of the campaign.

For campaign delivery in which no hang-up visits were conducted, the post-campaign use by under-fives was reduced to reflect the household survey results which found that children in households where all or some campaign nets were hung by a volunteer were more likely to use an LLIN. By reducing the proportion of children under five sleeping under an LLIN post-campaign in this scenario, the number of all-cause child deaths that could have been averted was lower and therefore the annual economic cost per child death averted increased by approximately 30%.

Therefore, although the inclusion of hang-up activities increases the cost per LLIN delivered (by USD 0.22 in Brong Ahafo and Western regions, and USD 0.29 in Central region), in all three regions the cost per death averted is lower and therefore more cost-effective. This suggests that a universal campaign with hang-up is the dominant strategy compared to a universal campaign without hang-up. It is important to note that this analysis is based on a number of key assumptions, particularly regarding the relative LLIN use by children under five depending on whether they live in households where volunteers did or did not hang campaign nets.

Recent data from operational research in Togo and Uganda suggests that there was no significant difference in LLIN use between households that received one or two additional follow-up visits after fixed-point LLIN distribution and those that did not receive any follow-up visits [21,22]. This appears to be in contrast to the findings here and some of the earlier mass campaigns which found that giving nets without their packaging and with follow-up ‘hang-up’ visits to households after distribution improved likelihood of LLIN use [23]. One suggestion is that post-distribution hang-up activities have less influence in countries or communities with an existing net culture. This may also help to explain the greater levels of post-campaign ownership and use in Brong Ahafo compared to Central and Western regions as these indicators were already higher in Brong Ahafo before the LLIN Campaign.

Therefore it is possible that a universal campaign that did not include house-to-house hang-up visits could still achieve the improvements in household ownership and use of LLINs seen following the LLIN Campaign. If this were the case, the cost per death averted for this alternative campaign design may actually be lower. However, the household survey analysis presented here suggests that household visits by volunteers were beneficial during the LLIN Campaign and that they should at least be considered in future mass campaign distributions in Ghana.

Unfortunately, it was not possible to directly compare the costs and effects of alternative campaign delivery designs within Ghana as the universal mass LLIN distribution was conducted in the same way across all ten regions. Although there are limitations to the basic economic modelling approach taken here compared to empirical data, sensitivity analyses have provided likely ranges around the cost effectiveness estimates and this has at least provided some insight in to the likely added value of the hang-up approach used in the context of Ghana.

## Conclusion

Overall, in terms of cost per LLIN delivered, the Ghana LLIN Campaign is comparable with previous mass campaigns and other LLIN distribution channels such as ANC and community-based distribution. Measures that increase the proportion of LLINs hanging in a household such as visits by volunteers and provision of hanging materials increased the likelihood that nets would be used in Brong Ahafo, Central and Western regions of Ghana. Although alternative campaign designs may be less costly, the risk that they would be less effective in improving LLIN use if hang-up visits were not included may reduce the cost effectiveness. This should be considered for future campaigns.

## Competing interests

The authors declare that they have no competing interests.

## Authors’ contributions

LSP, EA, MG, SA, VK, ER and AC devised the study design and objectives. LSP did the analysis and wrote the first draft of the manuscript. EA, SA, VK, CN, DS, AKM, MG, DY, ER, JA, ABW, CBP and LMJ contributed to data collection, analysis and interpretation. All authors read, commented on and approved the final manuscript.

## Supplementary Material

Additional file 1 Total financial cost and average annual economic cost of the LLIN campaign in Brong Ahafo, Central and Western regions. Click here for file

Additional file 2 Sensitivity of cost estimates to key assumptions. Click here for file
