# Supplementary material for: Evaluation of a universal long-lasting insecticidal net (LLIN) distribution campaign in Ghana: cost effectiveness of distribution and hang-up activities
Source: Malar J. 2014 Feb 28;13:71. doi: 10.1186/1475-2875-13-71 (PMC3944985; doi:10.1186/1475-2875-13-71)
Supplement: Additional file 1 — Total financial cost and average annual economic cost of the LLIN campaign in Brong Ahafo, Central and Western regions. [file 1475-2875-13-71-S1.pdf]

Additional File 1

Table S1: Total financial cost and average annual economic cost of the LLIN campaign in Brong Ahafo, Central and Western regions

	TOTAL FINANCIAL COST						AVERAGE ANNUAL ECONOMIC COST					
	**Brong Ahafo**	**%**	**Central**	**%**	**Western**	**%**	**Brong Ahafo**	**%**	**Central**	**%**	**Western**	**%**
**LLINs, Transport & Storage**	**5,469,317**	**74.5**	**6,071,091**	**79.3**	**8,158,505**	**82.3**	**1,933,570**	**60.3**	**2,146,315**	**64.4**	**2,885,412**	**70.3**
LLINs	4,993,733	68.0	5,623,752	73.5	7,551,223	76.1	1,765,436	55.0	1,988,167	59.6	2,69,586	65.1
Non-LLIN materials	357,351	4.9	412,228	5.4	539,160	5.4	126,334	3.9	145,735	4.4	190,610	4.7
Transport & storage	118,233	1.6	35,112	0.5	68,122	0.7	41,799	1.3	12,413	0.4	24,083	0.6
**Household Registration Phase**	**248,698**	**3.4**	**268,415**	**3.5**	**260,216**	**2.6**	**194,985**	**6.1**	**208,760**	**6.3**	**202,512**	**4.9**
Regional planning & training of trainers	39,493	0.6	45,121	0.6	43,787	0.5	18,464	0.6	21,908	0.7	21,436	0.5
Training of volunteers	38,293	0.5	40,994	0.5	47,189	0.5	26,881	0.8	26,373	0.8	27,132	0.7
Registration exercise	96,602	1.3	109,628	1.4	110,078	1.1	108,516	3.4	122,764	3.7	120,870	2.9
Supervision	74,310	1.0	72,672	0.9	59,162	0.6	41,124	1.3	37,715	1.1	33,074	0.8
**LLIN Distribution (including hang-up)**	**400,854**	**5.5**	**390,301**	**5.1**	**397,272**	**4.0**	**447,603**	**14.0**	**459,578**	**13.8**	**465,012**	**11.4**
Logistics training	57,701	0.8	59,236	0.8	58,931	0.6	25,149	0.8	25,226	0.8	25,118	0.6
Training of volunteers	116,236	1.6	139,377	1.8	120,316	1.2	69,478	2.2	78,792	2.4	72,821	1.8
Hang-Up exercise	-	-	-	-	-	-	223,092	7.0	252,022	7.6	256,689	6.3
Supervision	226,917	3.1	191,688	2.5	218,025	2.2	129,884	4.0	103,539	3.1	114,305	2.8
**Information, Education & Communication**	**222,225**	**3.0**	**220,970**	**2.9**	**180,018**	**1.8**	**119,581**	**3.7**	**143,535**	**4.3**	**97,998**	**2.4**
Social mobilization	156,192	2.1	116,634	1.5	119,569	1.2	57,262	1.8	43,150	1.3	43,359	1.1
Keep-up planning & supervision	18,481	0.3	17,604	0.2	13,212	0.1	18,481	0.6	17,604	0.5	13,212	0.3
IEC materials & activities	47,551	0.7	86,732	1.1	47,238	0.4	43,838	1.3	82,782	2.5	41,427	1.0
**Overheads**	**443,877**	**6.0**	**486,554**	**6.4**	629,721	**6.3**	**159,652**	**5.0**	**176,457**	**5.3**	**224,954**	**5.5**
**TOTAL PROVIDER COST**	**6,784,971**		**7,437,331**		**9,625,721**		**2,855,391**		**3,134,645**		**3,878,676**	
**Household-Level Costs**	**554,420**	**7.6**	**218,133**	**2.8**	**292,895**	**3.0**	**351,785**	**11.0**	**200,546**	**6.0**	**221,594**	**5.4**
Contribution to volunteers	554,420	7.6	218,133	2.8	292,895	3.0	196,004	6.1	77,117	2.3	103,547	2.5
Waiting time	-	-	-	-	-	-	155,780	4.9	123,429	3.7	118,047	2.9
**TOTAL SOCIETAL COST**	**7,339,391**		**7,655,464**		**9,918,628**		**3,207,176**		**3,335,191**		**4,100,270**	
