# Supplementary material for: Evaluation of a universal long-lasting insecticidal net (LLIN) distribution campaign in Ghana: cost effectiveness of distribution and hang-up activities
Source: Malar J. 2014 Feb 28;13:71. doi: 10.1186/1475-2875-13-71 (PMC3944985; doi:10.1186/1475-2875-13-71)
Supplement: Additional file 2 — Sensitivity of cost estimates to key assumptions. [file 1475-2875-13-71-S2.pdf]

Additional File 2

Table S2: Sensitivity of cost estimates to key assumptions (NOTE: all costs are economic costs from the societal perspective, presented in 2012 USD)

	**Brong Ahafo**				**Central**				**Western**			
	Cost per LLIN delivered	% deviation	Cost per death averted[1]	% deviation	Cost per LLIN delivered	% deviation	Cost per death averted[1]	% deviation	Cost per LLIN delivered	% deviation	Cost per death averted[1]	% deviation
Base case	2.42	-	5,026	-	3.35	-	7,499	-	3.06	-	8,702	-
Useful lifespan of LLIN												
2 years	3.55	47%	7,381	47%	4.90	46%	10,972	46%	4.50	47%	12,807	47%
5 years	1.51	38%	3,143	37%	2.11	37%	4,722	37%	1.91	38%	5,419	38%
Cost of LLIN												
USD 3.25	2.35	3%	4,895	3%	2.69	20%	6,022	20%	2.40	22%	6,830	22%
USD 4.80	3.00	24%	6,245	24%	3.39	1%	7,601	1%	3.10	1%	8,831	1%
No. child deaths averted per 1000 children sleeping under an LLIN												
3.4 per 1000	2.42	0%	8,130	62%	3.35	0%	12,131	62%	3.06	0%	14,076	62%
7.7 per 1000	2.42	0%	3,590	29%	3.35	0%	3,356	55%	3.06	0%	6,215	29%
Discount rate												
0%	2.28	6%	4,742	6%	3.16	6%	7,083	6%	2.89	6%	8,210	6%
10%	2.74	13%	5,708	14%	3.80	13%	8,502	13%	3.48	14%	9,885	14%

[1]*Additional all-cause child deaths averted by improvements in ITN use after the LLIN Campaign*
